# PAICS-Driven Purine Biosynthesis and Its Prognostic Implications in Lung Adenocarcinoma: A Novel Risk Stratification Model and Therapeutic Insights

**DOI:** 10.3390/cimb47050366

**Published:** 2025-05-16

**Authors:** Jinhui Liu, Qi-An Chen, Yannan Yang, Lin Zhang, Weihao Lin, Yuheng Hong, Yibo Gao

**Affiliations:** 1 Department of Thoracic Surgery, National Cancer Center/National Clinical Research Center for Cancer/Cancer Hospital, Chinese Academy of Medical Sciences and Peking Union Medical College, Beijing 100021, China; jinhliu0901@163.com (J.L.); b2022003038@student.pumc.edu.cn (Q.-A.C.); linwh1996@foxmail.com (W.L.); yhhong0701@163.com (Y.H.); 2 Department of Thoracic Surgery, National Cancer Center/National Clinical Research Center for Cancer/Cancer Hospital & Shenzhen Hospital, Chinese Academy of Medical Sciences and Peking Union Medical College, Shenzhen 518116, China; lance421320100@163.com (Y.Y.); zhanglinwhu@foxmail.com (L.Z.); 3 Central Laboratory & Shenzhen Key Laboratory of Epigenetics and Precision Medicine for Cancers, National Cancer Center/National Clinical Research Center for Cancer/Cancer Hospital & Shenzhen Hospital, Chinese Academy of Medical Sciences and Peking Union Medical College, Shenzhen 518116, China; 4 Laboratory of Translational Medicine, National Cancer Center/National Clinical Research Center for Cancer/Cancer Hospital, Chinese Academy of Medical Sciences and Peking Union Medical College, Beijing 100021, China; 5 State Key Laboratory of Molecular Oncology, National Cancer Center/National Clinical Research Center for Cancer/Cancer Hospital, Chinese Academy of Medical Sciences and Peking Union Medical College, Beijing 100021, China

**Keywords:** lung adenocarcinoma, purine metabolism, prognostic model, PAICS, tumor microenvironment

## Abstract

Background: Lung adenocarcinoma is the most common NSCLC and is associated with metabolic dysregulation. Purine biosynthesis, regulated by PAICS, plays a key role in tumor progression and therapy resistance. Methods: We focused on LUAD using pan-cancer and KEGG enrichment analyses. TCGA-LUAD and three GEO datasets were analyzed to confirm the prognostic relevance of purine biosynthesis. A prognostic model, the Purine Biosynthesis-Related Score (PBRS), was developed using LASSO regression and validated in independent cohorts. Gene set variation analysis, immune profiling, tumor mutational burden analysis, and drug sensitivity analysis were conducted. PAICS expression was validated in LUAD tissues, and its role was assessed via proliferation and migration assays. Results: PBRS classified LUAD patients into high-risk (PBRS-high) and low-risk (PBRS-low) subgroups, with distinct prognostic outcomes. PBRS-high patients showed enrichment in cell cycle regulation and DNA repair pathways and had higher TMB, suggesting potential sensitivity to immunotherapy, although immune escape mechanisms may limit the efficacy of immune checkpoint inhibitors. PBRS-low patients were more responsive to metabolic inhibitors. PAICS overexpression correlated with poor prognosis, while its knockdown suppressed LUAD progression. Conclusion: PBRS is a prognostic tool in LUAD, identifying PBRS-high patients who may benefit from immunotherapy or DDR-targeted therapies. PBRS-low patients exhibit sensitivity to metabolic inhibitors. PAICS is a potential therapeutic target.

## 1. Introduction

Lung cancer remains the leading cause of cancer-related deaths worldwide, accounting for approximately 2.5 million new cases and 1.8 million deaths in 2022 [1]. Among its subtypes, lung adenocarcinoma (LUAD) is the most prevalent and contributes to 7% of all cancer deaths [2,3]. Although chemotherapy, targeted therapy, and immunotherapy have improved treatment options, their efficacy remains limited due to drug resistance, low response rates, and tumor heterogeneity [4,5,6,7,8,9,10]. These limitations underscore the urgent need for novel prognostic tools and therapeutic strategies tailored to LUAD biology.

Metabolic reprogramming is a hallmark of cancer and plays a pivotal role in tumor growth and treatment response. Purine biosynthesis, maintained by both de novo and salvage pathways, is essential for nucleotide supply and supports rapid cell proliferation under metabolic stress [11,12,13]. Spatially coordinated through purinosome formation, this pathway is dynamically regulated by signaling nodes such as mTORC1 and ATF4 to maintain biosynthetic flux [14,15,16]. Recent studies have uncovered that perturbations in purine metabolism are associated with therapeutic resistance and immune evasion [13,17,18,19]. Notably, mitochondrial enzyme MTHFD2 promotes gefitinib-sensitive lung cancer cells developing resistance to gefitinib by sustaining purine production [17], while EGFR mutations upregulate salvage pathway enzymes like HPRT1 to maintain purine homeostasis [18].

Phosphoribosyl-aminocarboxymuconate semialdehyde (PAICS), a key enzyme in de novo purine biosynthesis (DNPB), catalyzes the conversion of AIR to SAICAR. Overexpression of PAICS has been linked to aggressive phenotypes in various cancers [20,21,22,23,24]. In LUAD, elevated PAICS expression correlates with poor prognosis [25,26], yet its mechanistic contributions and therapeutic potential remain largely unexplored.

In this study, we present a novel prognostic model—the Purine Biosynthesis-Related Score (PBRS)—developed from purine biosynthesis-related genes (PBRGs) and validated in both TCGA and GEO cohorts. PBRS serves as an independent predictor of overall survival and reveals tight associations with immune infiltration, tumor mutational burden (TMB), drug sensitivity, and key biological processes, including cell cycle regulation and DNA repair. Mechanistically, we highlight PAICS as a key metabolic driver that may promote tumor progression and immune modulation. This work provides the first integrated model linking purine biosynthesis to LUAD prognosis and treatment stratification, offering new insights into the metabolic-immune landscape of lung cancer.

## 2. Materials and Methods

### 2.1. Public Datasets Acquisition

**Figure 1:**
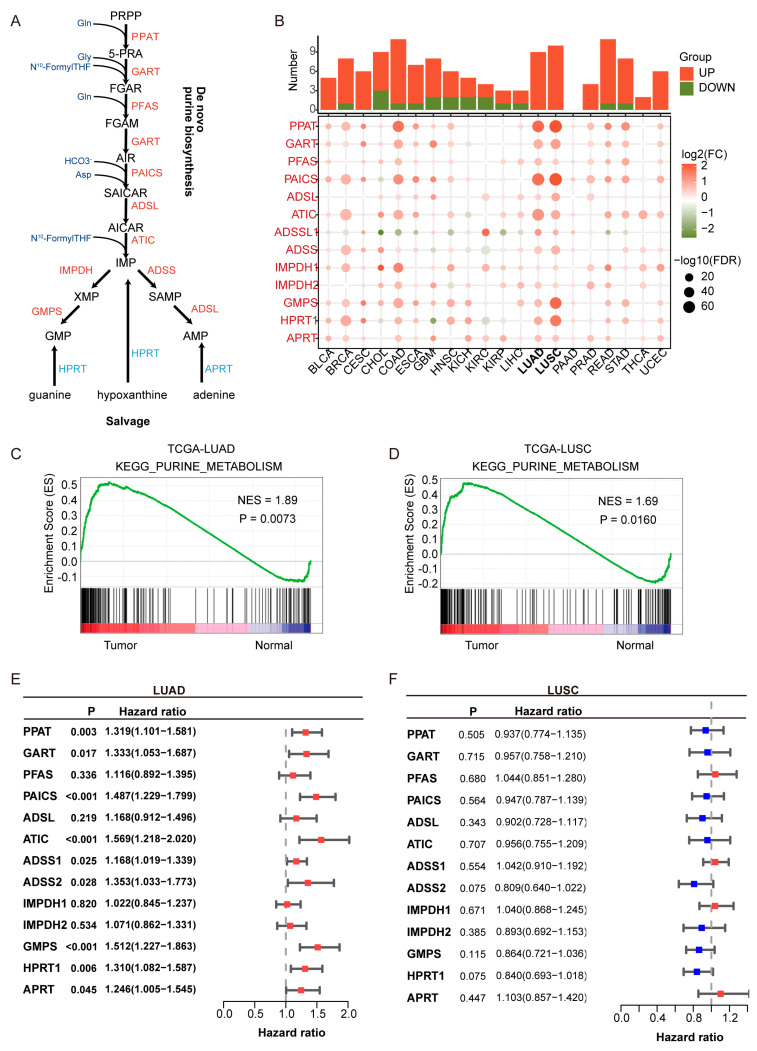
Upregulation and prognostic relevance of purine biosynthesis in lung adenocarcinoma (LUAD). (**A**) Schematic representation of the purine biosynthesis pathway, including both de novo and salvage branches. Enzymes highlighted in red are upregulated in LUAD. (**B**) Pan-cancer analysis of 14 purine biosynthesis-related genes (PBRGs) across 20 cancer types. The upper bar plot shows the number of significantly upregulated (red) and downregulated (green) genes. The dot plot below displays differential gene expression (log2FC) and statistical significance (−log10FDR). (**C**,**D**) KEGG enrichment analysis of the purine metabolism pathway in LUAD (**C**) and lung squamous cell carcinoma (LUSC) (**D**), showing elevated activity in tumors compared to normal tissues. NES: normalized enrichment score. (**E**,**F**) Forest plots of univariate Cox regression analyses showing the prognostic relevance of PBRGs in LUAD (**E**) and LUSC (**F**), based on hazard ratios (HRs) and 95% confidence intervals. Red bars (HR > 1) denote potential risk factors, while blue bars (HR < 1) denote potential protective factors.

Clinical information and transcriptomic profiles of patients with LUAD and LUSC were retrieved from The Cancer Genome Atlas (TCGA, Washington, DC, USA); https://portal.gdc.cancer.gov, accessed on 11 June 2021) and the Gene Expression Omnibus (GEO, Washington, DC, USA); http://www.ncbi.nlm.nih.gov/geo, accessed on 11 June 2021). The TCGA cohort served as the training set, while GEO-derived cohorts (GSE31210 (*n* = 226), GSE37745 [27], (*n* = 106) [28], and GSE50081 (*n* = 127) [29]) were utilized as external validation sets. Clinicopathological data of patients from public cohorts are summarized in Table S1. A total of 13 purine biosynthesis-related genes (PBRGs) were identified based on the purine biosynthesis pathway (Figure 1A, Table S2). These genes were selected for encoding key enzymes that catalyze rate-limiting or essential steps in both the de novo and salvage purine biosynthesis pathways, based on prior studies [13].

### 2.2. CHCAMS Cohort

In this study, a retrospective cohort comprising 195 LUAD patients was collected from the National Cancer Center. All patients in the CHCAMS cohort were pathologically confirmed LUAD cases, with available tumor tissue and corresponding clinical follow-up data. Patients who had received neoadjuvant chemotherapy or radiotherapy prior to surgery were excluded from the analysis. Tumor tissues and adjacent normal tissues were obtained from LUAD patients. Specifically, 15 paired samples were used for protein detection, 27 paired samples for RNA analysis, and 153 paired samples were incorporated into tissue microarrays (TMAs) for immunohistochemical (IHC) assays. Among the 195 patients, 149 had complete prognostic information, which was employed to assess survival status and construct prognostic survival curves.

### 2.3. Construction and Validation of the PSRS

To construct the Purine Biosynthesis-Related Score (PBRS), univariate Cox regression was first performed to identify genes significantly associated with overall survival. To avoid overfitting and enhance model robustness, we subsequently applied the least absolute shrinkage and selection operator (LASSO) regression. LASSO is particularly effective for high-dimensional biological data, as it penalizes regression coefficients and retains only variables that are strongly associated with the outcome. The optimal value of the regularization parameter (lambda) was selected using 10-fold cross-validation to minimize partial likelihood deviance. LASSO regression was applied to select PBRGs. The risk score for each sample was calculated using the following formula: RiskScore = (0.1379 × PAICS) + (0.0617 × ATIC) + (0.0655 × ADSS1) + (0.2127 × GMPS) + (0.0954 × APRT) + (0.0154 × HPRT1). The final PBRS model included six genes with non-zero coefficients. Patients in both the training and validation sets were stratified into PBRS-high and PBRS-low groups based on the optimal risk score cutoff. We chose the optimal risk score as the cutoff for PBRS-high and PBRS-low groups to maximize the predictive accuracy and clinical relevance of the stratification. This approach identifies the threshold that best differentiates between high- and low-risk patients, optimizing the prognostic value of the PBRS. By selecting the optimal cutoff, we ensure that the stratification reflects the most meaningful clinical distinctions, thus improving the precision of subsequent analyses. This method is commonly used in prognostic modeling to enhance the clinical applicability of the model and ensure that the stratification is driven by the data rather than arbitrary assumptions.

Kaplan–Meier survival curves and log-rank tests were performed using the R packages “survival” and “survminer” to evaluate survival outcomes. The prognostic predictive power was assessed via receiver operating characteristic (ROC) curves [30]. Univariate and multivariate Cox regression analyses were conducted to examine the independence of the risk score and clinical characteristics (age, stage, sex, and grade).

### 2.4. Exploration of Risk Score Correlationshanisms Related to the PBRS-Based Stratification in LUAD

#### 2.4.1. Differential Biological Processes

To elucidate the distinct biological processes associated with the Purine Biosynthesis-Related Score (PBRS) in lung adenocarcinoma (LUAD), we carried out two key analyses.

First, for the Gene Ontology (GO) enrichment analysis [31] of differentially expressed genes (DEGs) between the PBRS-high and PBRS-low groups, we utilized the “clusterProfiler” R package. This analysis aimed to identify the over-represented biological functions, molecular functions, and cellular components associated with the gene expression differences between the two risk subgroups.

Gene set variation analysis (GSVA) was performed using the ‘h.all.v7.2.symbols’ gene set from the Molecular Signatures Database (MSigDB), which includes 50 hallmark gene sets representing well-defined biological processes. This set was chosen for its broad coverage and minimal redundancy, making it ideal for interpreting major pathway alterations in cancer. Additionally, we incorporated predefined gene sets associated with immune phenotypes and checkpoint blockade response, as reported by Mariathasan et al. (2018) [32], to assess the immune landscape and potential immunotherapy response signatures. These gene sets have been validated across multiple tumor types and are widely used in immune-oncology research. Subsequently, GSVA [33] was performed using the ‘GSVA’ R package. We employed the “h.all.v7.2.symbols” gene set from the Molecular Signatures Database (MSigDB) and predefined gene sets from Mariathasan et al. The purpose of this GSVA was to estimate the enrichment scores of gene sets in each LUAD sample, thereby revealing the differences in the activity levels of various biological pathways between the PBRS-high and PBRS-low groups. These analyses together contribute to understanding the underlying biological mechanisms.

#### 2.4.2. Immune Correlation Analysis

The CIBERSORT algorithm was used to estimate the relative abundance of 22 immune cell types from bulk RNA-seq data. It applies a support vector regression-based approach to deconvolute immune cell composition using a reference signature matrix (LM22). However, CIBERSORT assumes linearity and requires high-quality expression data, which may limit its accuracy in cases of high tumor purity, batch effects, or stromal contamination. CIBERSORT was used to estimate immune cell infiltration abundance, and correlations between immune cell subsets and the risk signature were analyzed. Differences in immune checkpoint gene expression between PBRS-high and PBRS-low groups were evaluated. TMB data were downloaded, and Spearman correlation analysis was applied to assess the relationship between TMB and risk scores. Survival curves were constructed for four subgroups stratified by TMB and risk score.

#### 2.4.3. Drug Sensitivity Prediction

Drug sensitivity prediction was performed using the Genomics of Drug Sensitivity in Cancer (GDSC) database. While GDSC provides a valuable resource for high-throughput screening across various cancer cell lines, it does not fully replicate the complexity of in vivo tumor microenvironments or inter-patient heterogeneity. Therefore, predicted IC50 values should be interpreted cautiously when extrapolating to clinical drug responses. In this study, IC50 values were estimated using ridge regression models trained on GDSC cell line profiles. Lower predicted IC50 values indicate higher sensitivity, and differences in IC50 across PBRS subgroups were used to infer potential therapeutic vulnerabilities. Patient responses to chemotherapeutic agents were predicted using the Genomics of Drug Sensitivity in Cancer (GDSC, Hinxton, UK; https://www.cancerrxgene.org, accessed on 1 August 2021) database. The “pRRophetic” R package was employed to estimate the half-maximal inhibitory concentration (IC50) of each drug [34].

## 3. Results

### 3.1. Purine Metabolism Enrichment in LUAD and Its Association with Poor Prognosis

Purine metabolism is significantly upregulated in LUAD and is associated with poor prognosis. As shown in Figure 1A, key enzymes in purine de novo biosynthesis and salvage pathways were analyzed across 20 cancer types. Pan-cancer analysis revealed that purine biosynthesis enzymes, such as PPAT and PAICS, were significantly upregulated in LUAD and LUSC (Figure 1B), underscoring the critical role of purine metabolism in NSCLC. KEGG pathway enrichment analysis showed that the enrichment score (ES) for purine metabolism was higher in LUAD and LUSC compared to normal tissues, with a normalized enrichment score (NES) greater than 1 and a *p*-value < 0.05, indicating statistically significant enrichment.

Univariate Cox regression analysis indicated that all purine biosynthesis-related genes (PBRGs) exhibited hazard ratios (HRs) greater than 1 in LUAD (Figure 1E, Table S4), with nine genes (PPAT, GART, PAICS, ATIC, ADSS1, ADSS2, GMPS, HPRT1, APRT) significantly associated with overall survival (*p* < 0.05). In contrast, no PBRGs showed significant prognostic value in LUSC, highlighting limited clinical relevance in this subtype (Figure 1F, Table S5). These findings indicate that although both LUAD and LUSC exhibit activation of purine metabolism, LUAD may have distinct metabolic adaptation mechanisms, which are closely tied to its higher incidence, clinical features, and molecular characteristics. As a result, we focused our research on LUAD to ensure both clinical relevance and biological interpretability.

### 3.2. Construction of the Purine Biosynthesis-Related Score (PBRS) System for Overall Survival in LUAD Patients

**Figure 2:**
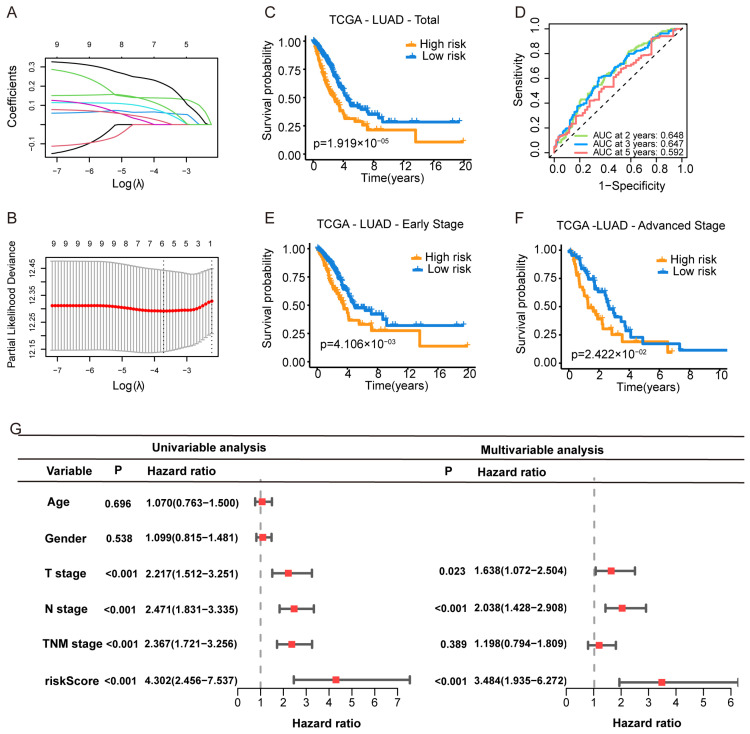
Construction of a Purine Biosynthesis-Related Score System (PBRS) in LUAD. (**A**) LASSO coefficient profiles of 13 candidate purine biosynthesis-related genes. (**B**) Ten-fold cross-validation for optimal λ selection in the LASSO regression model. (**C**) Kaplan–Meier curves showing OS stratified by PBRS in the TCGA-LUAD cohort. (**D**) Time-dependent ROC curves assessing model performance at 2, 3, and 5 years. (**E**,**F**) Kaplan–Meier curves of OS in early-stage (**E**) and advanced-stage (**F**) LUAD based on PBRS. (**G**) Forest plot showing univariate and multivariate Cox regression analyses incorporating age, sex, TNM stage, and PBRS in the TCGA cohort.

To construct a prognostic model based on purine biosynthesis-related genes (PBRGs), we performed LASSO regression analysis on 13 genes (Table S2). The final Purine Biosynthesis-Related Score (PBRS) model, which included six genes, was developed based on the optimal λ value (Figure 2A,B). The risk score was calculated using the following formula (Table S3):

PBRS = (0.1379 × PAICS) + (0.0617 × ATIC) + (0.0655 × ADSS1) + (0.2127 × GMPS) + (0.0954 × APRT) + (0.0154 × HPRT1)

Patients were stratified into PBRS-high and PBRS-low groups based on the optimal risk score. Kaplan–Meier survival analysis revealed that PBRS-high patients had significantly poorer overall survival (*p* = 1.9 × 10^−5^; Figure 2C). ROC analysis showed area under the curve (AUC) values for OS at 2, 3, and 5 years were 0.648, 0.647, and 0.592, respectively (Figure 2D). The model performed consistently across clinical stages, with PBRS-high patients demonstrating worse prognosis in both early (*p* = 4.1 × 10^−3^) and late stages (*p* = 2.4 × 10^−2^) of LUAD (Figure 2E,F). Survival analysis of individual genes (PAICS, ATIC, GMPS, ADSS1, APRT, and HPRT1) showed that high expression levels were associated with poor prognosis (Figure S1A–F).

To assess whether PBRS is an independent prognostic factor, univariate Cox regression analysis revealed that both PBRS and TNM stage were significantly associated with OS (PBRS: HR = 4.302, 95% CI = 2.456–7.537, *p* < 0.001; TNM stage: HR = 2.367, 95% CI = 1.721–3.256, *p* < 0.001; Figure 2G). Multivariate Cox regression confirmed PBRS as an independent prognostic indicator (HR = 3.484, 95% CI = 1.935–6.272, *p* < 0.001; Figure 2G).

Clinical characteristics associated with the PBRS-high and PBRS-low groups are summarized in Figure S2. PBRS-high patients exhibited a significantly higher mortality rate (*p* = 3.1 × 10^−5^) and more advanced clinical stages (*p* = 8.7 × 10^−4^). These findings suggest that PBRS can effectively predict prognosis and reflect disease severity in LUAD.

### 3.3. Validation of the PBRS

**Figure 3:**
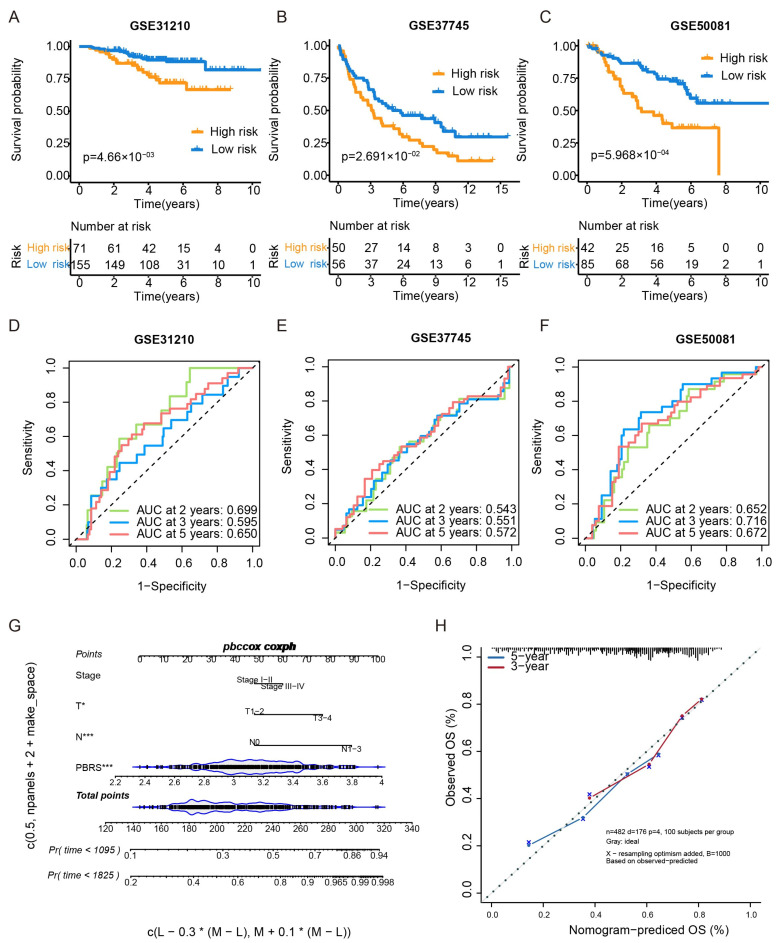
External validation of the PBRS model in GEO datasets and nomogram construction. (**A**–**C**) Kaplan–Meier survival curves comparing PBRS-high and PBRS-low groups in three independent GEO datasets (GSE31210, GSE37745, and GSE50081). (**D**–**F**) Time-dependent ROC curves evaluating the predictive performance of the PBRS model at 2, 3, and 5 years in the same cohorts. (**G**) Nomogram incorporating PBRS and TNM stage for predicting 1-, 3-, and 5-year OS in LUAD. (**H**) Calibration curves assessing the concordance between predicted and observed OS at 3 and 5 years. * *p* < 0.05; *** *p* < 0.001.

The prognostic performance of the PBRS was further validated in three independent GEO cohorts (GSE31210, GSE37745, and GSE50081). In all three datasets, patients in the PBRS-high group exhibited significantly poorer overall survival (Figure 3A–C). ROC curve analysis showed the following AUC values for OS:

GSE31210—2 years: 0.699, 3 years: 0.595, 5 years: 0.650 (Figure 3D).

GSE37745—2 years: 0.543, 3 years: 0.551, 5 yeas: 0.572 (Figure 3E).

GSE50081—2 years: 0.652, 3 years: 0.716, 5 years: 0.672 (Figure 3F).

Additionally, a nomogram integrating PBRS and TNM stage was constructed to enhance individualized survival prediction (Figure 3G). Calibration curves for 3- and 5-year survival demonstrated strong concordance between predicted and actual outcomes, highlighting the accuracy of the nomogram model (Figure 3H).

### 3.4. Correlation of PBRS with Biological Processes

**Figure 4:**
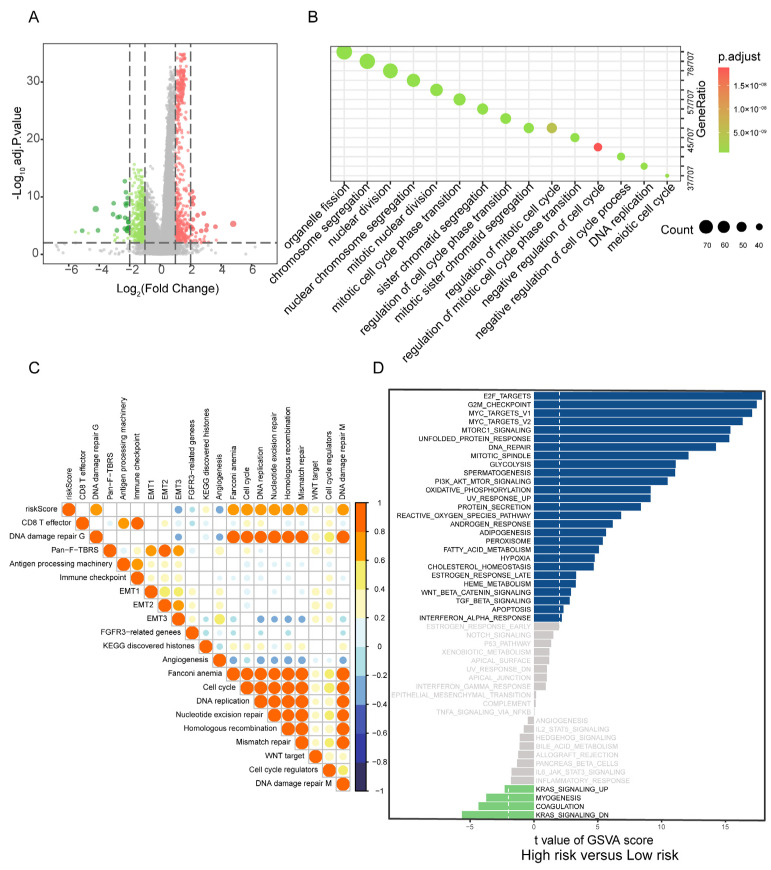
Correlation of PBRS with biological processes in LUAD. (**A**) Volcano plot of DEGs between PBRS-high and PBRS-low subgroups. Red dots represent significantly upregulated genes in the PBRS-high group, while green dots represent significantly downregulated genes. (**B**) GO enrichment analysis of DEGs, highlighting pathways related to cell cycle and DNA repair. (**C**) Spearman correlation analysis between the purine biosynthesis score and representative tumor-related gene signatures in LUAD. (**D**) GSVA showing differential activity of Hallmark pathways between PBRS-high and PBRS-low groups. The dashed line indicates a GSVA enrichment score of 0, serving as a baseline for distinguishing pathway upregulation and downregulation.

To explore the biological processes associated with PBRS, differentially expressed genes (DEGs) between PBRS-high and PBRS-low groups were identified, yielding a total of 17,743 genes (Figure 4A, Table S6). Pathway enrichment analysis revealed significant involvement in key oncogenic processes, including cell cycle regulation, DNA replication, DNA damage repair, and chromosome segregation (Figure 4B), all of which are essential for tumor cell proliferation and genomic maintenance.

Gene set variation analysis (GSVA) further demonstrated strong positive correlations between PBRS and tumor-promoting pathways, particularly those involved in cell cycle progression, nucleotide excision repair, homologous recombination, mismatch repair, and epithelial–mesenchymal transition (EMT1/EMT2) (Figure 4C).

Comparative GSVA between PBRS-high and PBRS-low subgroups highlighted distinct differences in pathway activity. The PBRS-high group exhibited enhanced activation of cell proliferation-related pathways, such as E2F targets, G2M checkpoint, and MYC targets, along with upregulation of PI3K-AKT-mTOR signaling and oxidative phosphorylation, indicating a highly proliferative and metabolically active tumor phenotype. In contrast, the PBRS-low group showed elevated activity in fatty acid metabolism and energy-efficient oxidative phosphorylation pathways, suggesting a less aggressive metabolic profile (Figure 4D).

### 3.5. PBRS Stratifies Immune Infiltration and TMB Heterogeneity in LUAD

**Figure 5:**
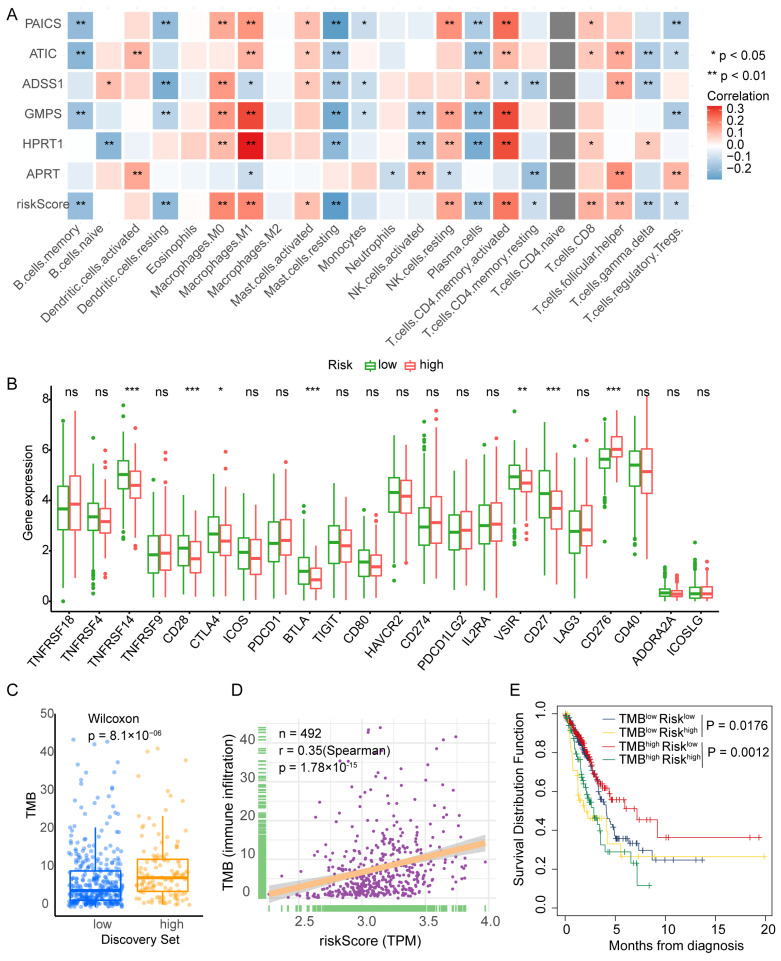
PBRS correlates with immune infiltration, TMB, and patient prognosis in LUAD. (**A**) CIBERSORT analysis of the correlation between PBRS, its component genes, and tumor-infiltrating immune cell populations. (**B**) Differential expression of immune-related genes between PBRS-high and PBRS-low groups. (**C**) Comparison of tumor mutational burden (TMB) between PBRS-high and PBRS-low subgroups. (**D**) Spearman correlation analysis between PBRS and TMB. (**E**) Kaplan–Meier survival curves for LUAD patients stratified by both PBRS and TMB. * *p* < 0.05; ** *p* < 0.01; *** *p* < 0.001.

To investigate the immune landscape associated with PBRS, tumor-infiltrating immune cell populations were estimated in TCGA-LUAD samples using the CIBERSORT algorithm. Correlation analysis revealed that PBRS was positively associated with M0 and M1 macrophages, resting natural killer (NK) cells, activated CD4^+^ T cells, follicular helper T cells (Tfh), and activated mast cells. In contrast, lower PBRS was linked to increased infiltration of memory B cells, resting dendritic cells, plasma cells, γδ T cells, regulatory T cells (Tregs), and resting CD4^+^ T cells (Figure 5A). These findings suggest that purine biosynthesis may shape the tumor immune microenvironment and influence antitumor immune responses.

Further analysis of immune checkpoint gene expression revealed that the PBRS-high group had reduced expression of TNFRSF14 (HVEM), CD27, CD28, BTLA, VSIR, and CTLA4, while CD276 (B7-H3) was upregulated (Figure 5B). These expression patterns suggest that purine biosynthesis may modulate immune checkpoint signaling in LUAD.

We next assessed tumor mutational burden (TMB) in relation to PBRS. The PBRS-high group exhibited significantly higher TMB (*p* = 8.1 × 10^−6^; Figure 5C), and Spearman correlation analysis confirmed a positive correlation between PBRS and TMB (r = 0.35, *p* = 1.78 × 10^−15^; Figure 5D). Moreover, in the high-TMB subgroup, patients with high PBRS showed significantly worse survival outcomes (*p* = 1.2 × 10^−3^; Figure 5E), indicating that PBRS may further stratify prognosis among TMB-high patients.

**Figure 6:**
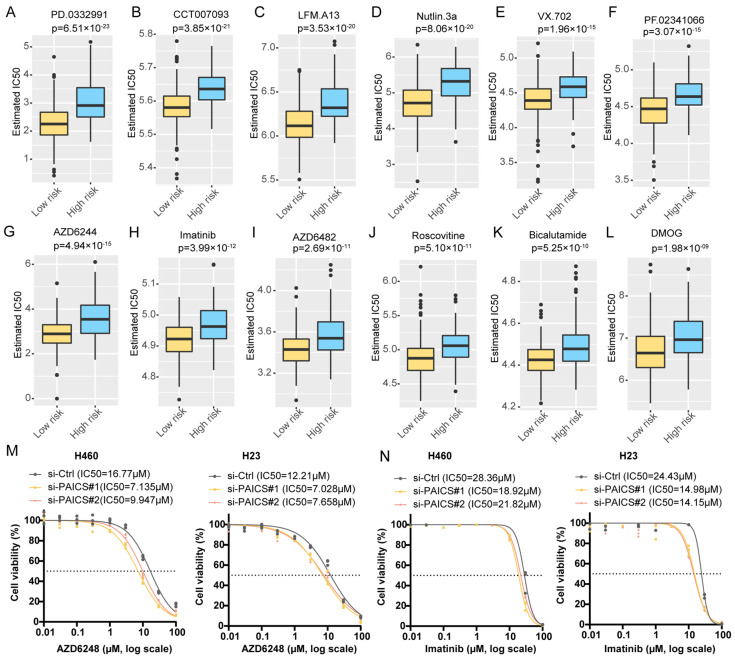
Differential predicted IC50 values in PBRS risk stratification. Differential IC50 values for targeted drugs in PBRS-high and PBRS-low groups, showing higher sensitivity in PBRS-low patients. (**A**) PD.0332991 (*p* = 6.51 × 10^−23^). (**B**) CCT007093 (*p* = 3.85 × 10^−21^). (**C**) LFM.A13 (*p* = 3.53 × 10^−20^). (**D**) Nutlin.3a (*p* = 8.06 × 10^−20^). (**E**) VX.702 (*p* = 1.96 × 10^−15^). (**F**) PF.02341066 (*p* = 3.07 × 10^−15^). (**G**) AZD6244 (*p* = 4.94 × 10^−15^). (**H**) Imatinib (*p* = 3.99 × 10^−12^). (**I**) AZD6482 (*p* = 2.69 × 10^−11^). (**J**) Roscovitine (*p* = 5.10 × 10^−11^). (**K**) Bicalutamide (*p* = 5.25 × 10^−10^). (**L**) DMOG (*p* = 1.98 × 10^−09^). (**M**,**N**) Drug sensitivity curves for AZD6482 (**M**) and imatinib (**N**) in the H460 and H23 cell lines following PAICS knockdown. The dashed lines indicate the IC50 values used as a reference to compare drug response between groups.

To explore therapeutic implications, we estimated the half-maximal inhibitory concentration (IC50) of 138 compounds using drug sensitivity data from the Genomics of Drug Sensitivity in Cancer (GDSC) database. Compared to PBRS-high patients, those in the PBRS-low group were more likely to benefit from agents such as the MEK inhibitor AZD6482, MDM2 inhibitor Nutlin-3a, tyrosine kinase inhibitor imatinib, and CDK inhibitor Roscovitine (Figure 6A–L, Table S7). Additionally, we experimentally evaluated the IC50 values of AZD6482 and imatinib in the H460 and H23 cell lines following PAICS knockdown. PAICS suppression significantly reduced the IC50 values of both AZD6482 (Figure 6M) and imatinib (Figure 6N), suggesting that reduced PAICS expression enhances cellular sensitivity to these agents.

### 3.6. PAICS as a Key Driver of LUAD Malignancy

**Figure 7:**
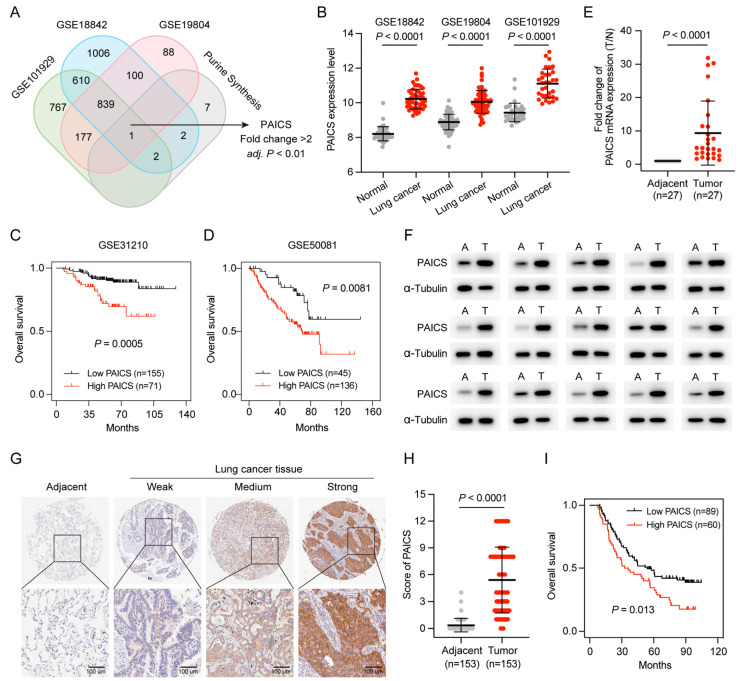
High PAICS expression is associated with poor prognosis in GEO and CHCAMS cohorts. (**A**) Venn diagram of PBRGs across three GEO datasets, highlighting PAICS as the only consistently upregulated gene. (**B**) PAICS expression levels between LUAD and adjacent normal tissues in the GEO datasets. (**C**,**D**) Kaplan–Meier survival analysis based on PAICS expression in GEO cohorts. (**E**,**F**) Validation of PAICS mRNA and protein expression in the CHCAMS cohort by qPCR and Western blot. (**G**,**H**) Representative IHC images and quantification of PAICS expression in LUAD versus normal tissues in the CHCAMS cohort. (**I**) Kaplan–Meier survival curves stratified by PAICS expression in the CHCAMS cohort. Data represent mean ± SD (*n* = 3); results are from triplicate experiments.

Purine nucleotides are essential for cell proliferation, and PAICS, a key enzyme in de novo purine synthesis, was identified as a potential regulator in LUAD. To further investigate its role, purine metabolism–related genes were intersected across three datasets (GSE101929, GSE18842, and GSE19804), with PAICS emerging as the only gene consistently associated with purine metabolism (fold change > 2, *p* < 0.01; Figure 7A).

PAICS expression was significantly elevated in LUAD compared to adjacent normal tissues (Figure 7B), and Kaplan–Meier analysis showed that high PAICS expression was associated with worse OS (Figure 7C,D).

PAICS expression was further validated in the CHCAMS cohort using qPCR, Western blot, and IHC. Both RNA and protein levels were significantly upregulated in LUAD tissues relative to normal controls (Figure 7E,F). IHC confirmed strong PAICS staining in LUAD samples (*p* < 0.0001; Figure 7G,H), and high expression was significantly associated with poor prognosis (*p* < 0.05; Figure 7I).

**Figure 8:**
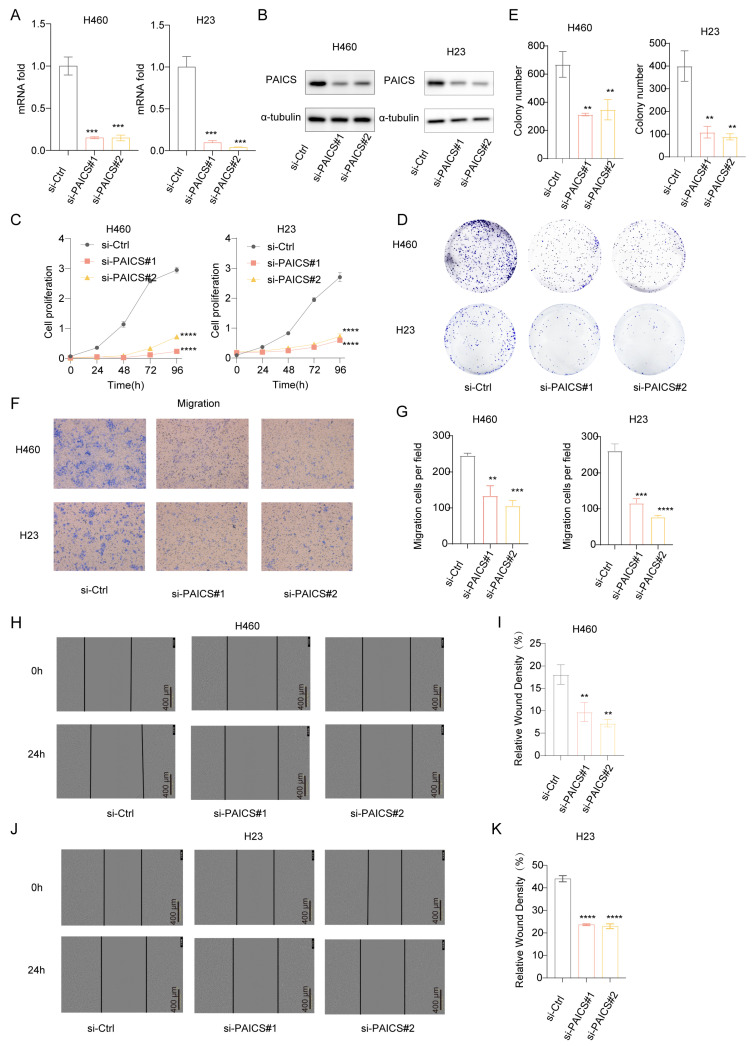
PAICS knockdown inhibits proliferation and migration of H460/H23 lung cancer cells in vitro. siRNA targeting PAICS was transfected into H460 and H23 cells. (**A**) mRNA levels of PAICS after siRNA transfection. (**B**) Protein levels of PAICS after siRNA transfection. (**C**) Cell proliferation assay (CCK-8) showing growth curves over 96 h (*n* = 5 technical replicates per group). (**D**,**E**) Colony formation assay: (**D**) representative images of crystal violet-stained colonies; (**E**) quantification of colony number. (**F**,**G**) Transwell migration assay: (**F**) representative images of migrated cells; (**G**) quantification of cell counts. (**H**–**K**) Wound healing assay: (**H**,**I**) H460 cells and (**J**,**K**) H23 cells at 0 and 24 h post-scratch. (**H**,**J**) Representative brightfield images. (**I**,**K**) Quantification of wound closure rate (scale bar = 600 μm). Results are from triplicate experiments, Data represent mean ± SD (*n* = 3). ** *p* < 0.01, *** *p* < 0.001, **** *p* < 0.0001.

To investigate the functional role of PAICS in LUAD, we conducted both knockdown and overexpression experiments. In H460 and H23 cell lines, PAICS expression was efficiently silenced using si-PAICS#1 and si-PAICS#2, as confirmed by reduced mRNA and protein levels (Figure 8A,B). PAICS knockdown significantly suppressed cell proliferation (Figure 8C), decreased clonogenic capacity (Figure 8D,E), and impaired migratory ability (Figure 8F–K).

**Figure 9:**
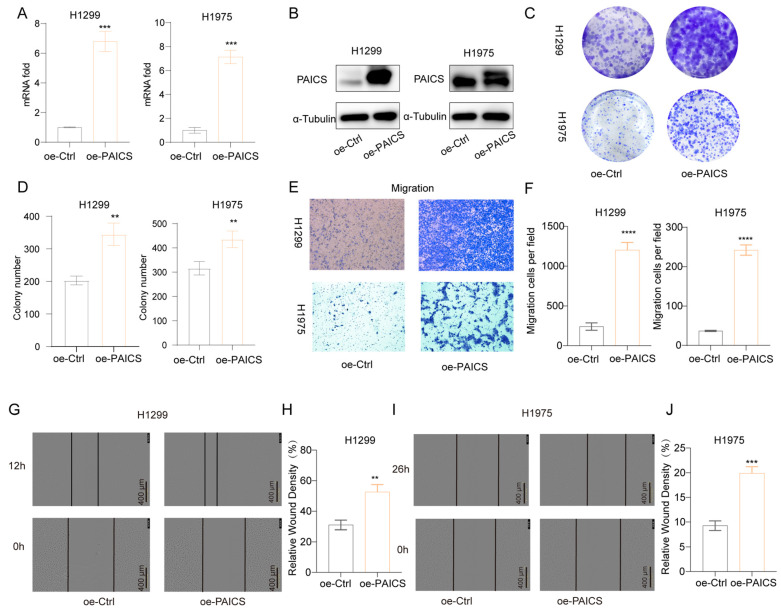
PAICS overexpression enhances malignant phenotypes in H1299 and H1975 cells (**A**) qRT-PCR validation of PAICS overexpression. (**B**) Western blot analysis of PAICS protein levels. (**C**,**D**) Colony formation assay: (**C**) Representative images of crystal violet-stained colonies; (**D**) Quantification of colony number. (**E**,**F**) Transwell migration assay: (**E**) Representative images of migrated; (**F**) Quantification of cell counts. (**G**–**J**) Wound healing assay: (**G**,**H**) H1299 cells at 0 and 12 h post-scratch and (**I**,**J**) H1975 cells at 0 and 16 h post-scratch. (**G**,**I**) Representative brightfield images; (**H**,**J**) Quantification of wound closure rate (scale bar = 600 μm). Results are from triplicate experiments. Data represent mean ± SD (*n* = 3). ** *p* < 0.01, *** *p* < 0.001, **** *p* < 0.0001.

Conversely, overexpression of PAICS in H1299 and H1975 cells led to increased PAICS mRNA and protein expression (Figure 9A,B), which promoted clonogenic growth (Figure 9C,D), enhanced cell migration (Figure 9E,F), and accelerated wound closure in scratch assays (Figure 9G–J).

Collectively, these results demonstrate that PAICS plays a pivotal role in regulating LUAD cell proliferation, clonogenicity, migration, and invasion, underscoring its potential as a key driver of tumor progression and a promising therapeutic target.

## 4. Discussion

Lung adenocarcinoma (LUAD), the most prevalent subtype of non-small cell lung cancer (NSCLC), remains a leading cause of cancer-related mortality, with a poor prognosis and a five-year survival rate below 30% due to late-stage diagnosis, chemotherapy resistance, and high recurrence rates [1,4,5,6,7,8,9,10]. Emerging evidence highlights the central role of purine biosynthesis in tumor progression and therapy resistance. Purine biosynthesis not only provides nucleotides necessary for rapid tumor cell proliferation but also modulates drug responses and influences the tumor microenvironment [17,18,35,36,37,38,39,40,41,42]. In this study, we developed and validated a prognostic signature, the Purine Biosynthesis-Related Score (PBRS), based on six core purine biosynthesis-related genes (PBRGs)—PAICS, ATIC, ADSS1, GMPS, APRT, and HPRT1. This model demonstrates significant prognostic value, with PBRS-high patients exhibiting worse overall survival (OS) compared to PBRS-low patients. Furthermore, multivariate Cox regression confirmed PBRS as an independent prognostic factor, and external validation using the GSE31210, GSE37745, and GSE50081 datasets supported the robustness of PBRS in predicting LUAD prognosis. Interestingly, the predictive performance of PBRS varied across the different datasets. Specifically, GSE31210 and GSE50081 demonstrated higher AUC values, likely due to their homogeneous clinical characteristics and a higher prevalence of early-stage disease. In contrast, the GSE37745 dataset showed a lower AUC, which could be attributed to incomplete clinical data and a higher mortality rate among patients. These variations in predictive performance highlight the cohort-dependent nature of the PBRS model but also underscore its overall robustness and potential applicability across diverse LUAD patient populations.

Our analysis of differentially expressed genes (DEGs) between PBRS-high and PBRS-low groups through Gene Ontology (GO) and gene set variation analysis (GSVA) revealed significant enrichment in key carcinogenic pathways, such as cell cycle regulation, DNA replication, DNA damage repair, epithelial–mesenchymal transition (EMT), and chromosome segregation. These pathways, which are closely tied to purine biosynthesis, suggest that enhanced purine metabolism supports tumor cell proliferation and contributes to aggressive tumor behavior in PBRS-high tumors. Notably, PAICS and ATIC regulate critical steps in the G1-S transition and DNA replication, thus supporting rapid tumor growth [23,24,43].

In PBRS-high tumors, DNA repair pathways are upregulated as a compensatory response to replication stress, a hallmark of rapid tumor cell proliferation [44]. PAICS, a key enzyme in purine biosynthesis, plays a crucial role in DNA damage repair by interacting with histone deacetylases (HDAC1/2) to regulate DAD51 expression, enhancing the repair of DNA damage sites [45]. Importantly, PAICS deficiency impairs DNA repair and increases tumor cell sensitivity to DNA-damaging agents like cisplatin [45]. In addition, inhibition of ATIC activates cell cycle checkpoints, especially the G2/M phase, making PBRS-high cells more sensitive to radiation [43]. These findings suggest that DDR (DNA damage repair) inhibition could be a promising therapeutic strategy for PBRS-high LUAD patients. Inhibition of DDR has been shown to enhance the efficacy of radiotherapy, chemotherapy, and DDR-targeted agents, such as the PARP inhibitor olaparib, improving clinical outcomes in cancer patients [46,47]. The combination of DDR inhibitors with immune checkpoint inhibitors (ICIs) has shown potential [48], particularly for patients with high PBRS scores, suggesting a dual benefit of DDR-targeted therapies in these patients.

Furthermore, the EMT-related markers identified in PBRS-high tumors highlight a more invasive phenotype. PAICS facilitates FAK phosphorylation, which is involved in promoting EMT in cancer cells [49]. This further supports the role of purine biosynthesis in tumor progression. Similarly, the activation of chromosome segregation pathways points to genomic instability, a key hallmark of tumor progression, often associated with increased resistance to treatment and poor prognosis [50].

The activation of hallmark gene sets such as E2F targets, the G2/M checkpoint, and MYC target genes further supports that tumors with high PBRS expression exhibit elevated proliferative potential and DNA repair activity. Enhanced purine biosynthesis provides the nucleotides needed for rapid DNA replication, promoting E2F-driven cell cycle progression and G2/M checkpoint activation. MYC, as a master regulator of metabolism and proliferation, upregulates purine biosynthesis enzymes like PAICS and ATIC, boosting nucleotide synthesis [51,52]. MYC also stimulates E2F target genes, promoting cell cycle progression and increasing CDK2/Cyclin E activity. Additionally, PAICS knockdown blocks the G1-S transition, partly by inhibiting Cyclin E and upregulating Cyclin D1, P21, and CDK4. Thus, purine metabolism directly mediates tumor proliferation and development through MYC target genes [53]. MYC enhances purine biosynthesis, increasing nucleotide supply and facilitating E2F-driven cell cycle progression, ensuring DNA replication is completed and undamaged.

Recent advances in immunotherapy have reshaped the treatment landscape for NSCLC [54], yet the heterogeneous efficacy of these therapies highlights the need for reliable predictive biomarkers. In this study, we examined how purine biosynthesis impacts the tumor immune microenvironment by analyzing the relationship between PBRS and immune cell infiltration. We found that PBRS-high tumors exhibited an altered immune profile, with increased infiltration of M0/M1 macrophages, activated CD4^+^ T cells, and NK cells. These findings are biologically significant as they indicate that the upregulation of purine biosynthesis may help establish a more immune-activated microenvironment. Specifically, M1 macrophages and activated CD4^+^ T cells are generally associated with anti-tumor immunity, promoting immune responses against the tumor [55]. NK cell infiltration further suggests that the tumor is undergoing enhanced innate immune surveillance, potentially increasing the likelihood of immune system detection and response [55]. However, increased purine metabolism may disrupt immune balance by remodeling immune checkpoint expression profiles. PBRS-high tumors exhibit downregulation of co-stimulatory molecules like HVEM, CD27, CD28, and BTLA, while immune-suppressive CD276 (B7-H3) is upregulated. This supports previous studies linking CD28 expression with favorable responses to ICB [56], while B7-H3 promotes immune escape by inhibiting T/NK cell functions [57]. Notably, downregulation of the BTLA-HVEM axis weakens immune activation signals, especially in PD-L1-negative NSCLC patients, where its expression correlates with prognosis [58].

Interestingly, although the PBRS-high expression group exhibited a higher tumor mutational burden (TMB)—a marker typically associated with better ICI response—its clinical outcomes were paradoxically worsened. Mechanistically, loss of co-stimulatory signals like HVEM/CD28 could impair antigen presentation of new tumor antigens generated by high TMB, while the immune-suppressive microenvironment mediated by B7-H3 may counterbalance the immune recognition advantage from genomic instability. Recent studies also highlight that NSCLC cells activate the purine–PD-L1 axis through CXCL8-mediated macrophage recruitment, and glutamine antagonist DRP-104 can enhance ICI efficacy by inhibiting purine biosynthesis [19], providing experimental evidence for the metabolic-immune regulatory mechanisms discussed.

Purine biosynthesis metabolism has been shown in existing research to enhance effector immune cell function through two mechanisms: (1) by directly supporting T-cell energy metabolism and nucleotide synthesis [59], and (2) by contributing to immune escape through metabolic dysregulation in tumor cells and immune-suppressive populations [42,60,61]. Tumor-infiltrating CD8^+^ T cells exhibit higher purine biosynthesis activity than their counterparts in peripheral immune organs, and supplementation with one-carbon units has been shown to further enhance their anti-tumor capabilities [59]. This suggests that targeting purine biosynthesis metabolism could potentially boost immune responses during immunotherapy. However, aberrant activation of purine biosynthesis in tumor cells could contribute to immune checkpoint inhibitor (ICI) resistance by competing for metabolic resources or by releasing immune-suppressive signals, as seen in other cancers [42]. This existing body of work supports our findings by highlighting the metabolic influence on immune response and treatment efficacy.

We assessed drug sensitivity in PBRS groups using IC_50_ estimates for 138 compounds from the Genomics of Drug Sensitivity in Cancer (GDSC) database. PBRS-low patients showed increased sensitivity to agents like AZD6482 and imatinib, likely due to their effects on tumor metabolism and immune characteristics. AZD6482, a selective PI3Kβ inhibitor, blocks Akt signaling and tumor growth in cells with p110β activation or PTEN loss [62]. In addition to its signaling role, PI3Kβ modulates cellular metabolism through O-GlcNAcylation and acetyl-CoA production [63]. In PBRS-low tumors, AZD6482′s inhibition of the PI3K/Akt pathway increases metabolic stress, reduces proliferation, and enhances therapeutic vulnerability. Similarly, imatinib, a tyrosine kinase inhibitor, downregulates purine biosynthesis, imposing additional metabolic pressure [64]. Multi-omics studies show that imatinib reduces purine metabolism gene expression and nucleotide metabolite levels, further increasing metabolic stress [64]. Combining imatinib with 1C metabolism inhibition has been shown to reduce drug-resistant leukemia stem cells [65], suggesting its efficacy in LUAD. These findings suggest PBRS-low patients may benefit more from metabolic targeting therapies like AZD6482 and imatinib, offering a personalized treatment approach for LUAD. These drugs disrupt signaling pathways linked to nucleotide synthesis and cell cycle regulation. For example, MEK inhibitors impair purine biosynthesis indirectly by reducing MYC-driven transcription [52], while CDK inhibitors restrict cell cycle progression, which is dependent on nucleotide availability. Therefore, PBRS may serve as both a prognostic biomarker and a predictive indicator for selecting targeted therapies.

The PBRS model consists of six core PBRGs: PAICS, ATIC, ADSS1, GMPS, APRT, and HPRT1. PAICS, ATIC, ADSS1, and GMPS are involved in de novo purine biosynthesis (DNPB). PAICS enhances DNPB flux by recruiting UBAP2, boosting nucleotide synthesis and tumor proliferation [66]. ATIC, regulated by MYC, supports nucleotide metabolism and proliferation [67]. ADSS1 is a novel LUAD target [68], while GMPS stabilizes p53 via USP7 interaction [69]. APRT and HPRT1, key enzymes in purine salvage, with APRT require further study to fully understand its role [70]. HPRT1 also promotes EMT and tumor proliferation through STAT3 interaction [71].

We sought to understand the reasons behind the upregulation of purine biosynthesis-related genes (PBRGs) in LUAD. ERK2-mediated PFAS phosphorylation promotes nucleic acid synthesis [72]. Hypoxia enhances purine biosynthesis enzyme expression via HIF-1α activation [18], while L-glutamine regulates PPAT and PAICS activity to support cell proliferation [26]. These findings highlight purine biosynthesis upregulation as a central feature of metabolic reprogramming in LUAD, sustaining tumor growth and providing potential therapeutic targets.

PAICS was identified as a core gene in purine biosynthesis continuously associated with LUAD through multi-cohort database screening. Functional validation confirmed that PAICS knockdown inhibited tumor cell proliferation and migration, and increased sensitivity to AZD6482 and imatinib. PAICS’ role in purinosome assembly and its regulation of nucleotide synthesis and tumor proliferation makes it a potential therapeutic target. Future studies should focus on developing small-molecule inhibitors targeting PAICS or its downstream signaling pathways, as well as exploring combination therapies with traditional treatments like chemotherapy and immunotherapy to enhance therapeutic efficacy.

Despite the promising predictive value of PBRS, its direct implementation into clinical practice warrants further discussion. Given that PBRS is based on transcriptomic profiling, integrating it into routine diagnostics would require standardized RNA sequencing or qRT-PCR-based platforms. With advancements in precision oncology, these technologies are increasingly feasible. Additionally, a PBRS-based gene panel compatible with formalin-fixed, paraffin-embedded (FFPE) tissues could be developed for easier adoption in pathology laboratories. However, several limitations remain, such as cohort-dependent variability, technical inconsistencies in gene expression quantification, and the lack of prospective validation in large-scale, multi-center clinical studies. Moreover, PBRS does not incorporate clinical parameters like comorbidities, histologic variants, or performance status, which may influence clinical decision making. Future research should focus on validating PBRS in prospective cohorts, evaluating its cost-effectiveness, reproducibility, and its utility in real-world clinical settings.

In summary, this study underscores the prognostic and therapeutic potential of PBRS in LUAD. PBRS is a robust tool for stratifying LUAD patients and predicting response to DDR-targeted therapies, particularly in combination with immunotherapy. Despite the promising findings, several limitations must be considered. First, the retrospective nature of the analysis using public datasets may introduce selection bias. Second, the clinical implementation of PBRS requires standardization of RNA-based assays, and its validation in prospective cohorts is needed. Third, the understanding of PAICS’s mechanistic role is based primarily on in vitro models, and drug sensitivity experiments lack in vivo validation. Additionally, its applicability to other cancer types and potential limitations in clinical practice, such as the need for large-scale validation and challenges in integrating it into routine clinical workflows, must also be addressed. Future studies should focus on developing small-molecule inhibitors targeting PAICS or its downstream signaling pathways, as well as exploring the combination of PAICS inhibition with traditional treatments like chemotherapy or immunotherapy to enhance therapeutic efficacy. Additionally, prospective validation of PBRS is needed to establish its utility as a clinical tool for patient stratification and treatment decision making. Developing preclinical models targeting PAICS and investigating its role in DNA damage repair and immune modulation could open new therapeutic avenues for LUAD treatment, ultimately improving patient outcomes and therapeutic efficacy.

## Data Availability

The data presented in this study are available upon reasonable request from the corresponding author.

## Supplementary Materials

The following supporting information can be downloaded at: https://www.mdpi.com/article/10.3390/cimb47050366/s1.

## References

[1] Bray F., Laversanne M., Sung H., Ferlay J., Siegel R.L., Soerjomataram I., Jemal A. (2024). Global Cancer Statistics 2022: GLOBOCAN Estimates of Incidence and Mortality Worldwide for 36 Cancers in 185 Countries. CA Cancer J. Clin..

[2] Mazzilli S.A., Rahal Z., Rouhani M.J., Janes S.M., Kadara H., Dubinett S.M., Spira A.E. (2025). Translating Premalignant Biology to Accelerate Non-Small-Cell Lung Cancer Interception. Nat. Rev. Cancer.

[3] Li Z., Zhuang X., Pan C.-H., Yan Y., Thummalapalli R., Hallin J., Torborg S., Singhal A., Chang J.C., Manchado E. (2024). Alveolar Differentiation Drives Resistance to KRAS Inhibition in Lung Adenocarcinoma. Cancer Discov..

[4] Basil M.C., Katzen J., Engler A.E., Guo M., Herriges M.J., Kathiriya J.J., Windmueller R., Ysasi A.B., Zacharias W.J., Chapman H.A. (2020). The Cellular and Physiological Basis for Lung Repair and Regeneration: Past, Present, and Future. Cell Stem Cell.

[5] Antonia S.J., Villegas A., Daniel D., Vicente D., Murakami S., Hui R., Kurata T., Chiappori A., Lee K.H., De Wit M. (2018). Overall Survival with Durvalumab after Chemoradiotherapy in Stage III NSCLC. N. Engl. J. Med..

[6] Spigel D.R., Faivre-Finn C., Gray J.E., Vicente D., Planchard D., Paz-Ares L., Vansteenkiste J.F., Garassino M.C., Hui R., Quantin X. (2022). Five-Year Survival Outcomes from the PACIFIC Trial: Durvalumab After Chemoradiotherapy in Stage III Non-Small-Cell Lung Cancer. J. Clin. Oncol..

[7] Camidge D.R., Pao W., Sequist L.V. (2014). Acquired Resistance to TKIs in Solid Tumours: Learning from Lung Cancer. Nat. Rev. Clin. Oncol..

[8] Qian X., Guo X., Li T., Hu W., Zhang L., Wu C., Ye F. (2022). Efficacy of Immune Checkpoint Inhibitors in EGFR-Mutant NSCLC Patients with EGFR-TKI Resistance: A Systematic Review and Meta-Analysis. Front. Pharmacol..

[9] Hastings K., Yu H.A., Wei W., Sanchez-Vega F., DeVeaux M., Choi J., Rizvi H., Lisberg A., Truini A., Lydon C.A. (2019). EGFR Mutation Subtypes and Response to Immune Checkpoint Blockade Treatment in Non-Small-Cell Lung Cancer. Ann. Oncol..

[10] Tang Y., Fang W., Zhang Y., Hong S., Kang S., Yan Y., Chen N., Zhan J., He X., Qin T. (2015). The Association between PD-L1 and EGFR Status and the Prognostic Value of PD-L1 in Advanced Non-Small Cell Lung Cancer Patients Treated with EGFR-TKIs. Oncotarget.

[11] Yin J., Ren W., Huang X., Deng J., Li T., Yin Y. (2018). Potential Mechanisms Connecting Purine Metabolism and Cancer Therapy. Front. Immunol..

[12] Mullen N.J., Singh P.K. (2023). Nucleotide Metabolism: A Pan-Cancer Metabolic Dependency. Nat. Rev. Cancer.

[13] Tran D.H., Kim D., Kesavan R., Brown H., Dey T., Soflaee M.H., Vu H.S., Tasdogan A., Guo J., Bezwada D. (2024). De Novo and Salvage Purine Synthesis Pathways across Tissues and Tumors. Cell.

[14] French J.B., Jones S.A., Deng H., Pedley A.M., Kim D., Chan C.Y., Hu H., Pugh R.J., Zhao H., Zhang Y. (2016). Spatial Colocalization and Functional Link of Purinosomes with Mitochondria. Science.

[15] Ben-Sahra I., Hoxhaj G., Ricoult S.J.H., Asara J.M., Manning B.D. (2016). mTORC1 Induces Purine Synthesis through Control of the Mitochondrial Tetrahydrofolate Cycle. Science.

[16] Wu Z., Bezwada D., Cai F., Harris R.C., Ko B., Sondhi V., Pan C., Vu H.S., Nguyen P.T., Faubert B. (2024). Electron Transport Chain Inhibition Increases Cellular Dependence on Purine Transport and Salvage. Cell Metab..

[17] Nishimura T., Nakata A., Chen X., Nishi K., Meguro-Horike M., Sasaki S., Kita K., Horike S., Saitoh K., Kato K. (2019). Cancer Stem-like Properties and Gefitinib Resistance Are Dependent on Purine Synthetic Metabolism Mediated by the Mitochondrial Enzyme MTHFD2. Oncogene.

[18] Geng P., Ye F., Dou P., Hu C., He J., Zhao J., Li Q., Bao M., Li X., Liu X. (2024). HIF-1α-HPRT1 Axis Promotes Tumorigenesis and Gefitinib Resistance by Enhancing Purine Metabolism in EGFR-Mutant Lung Adenocarcinoma. J. Exp. Clin. Canc. Res..

[19] Yang L., Li A., Yu W., Wang H., Zhang L., Wang D., Wang Y., Zhang R., Lei Q., Liu Z. (2025). Blockade of Purine Metabolism Reverses Macrophage Immunosuppression and Enhances Anti-Tumor Immunity in Non-Small Cell Lung Cancer. Drug Resist. Updates.

[20] Huo A., Xiong X. (2023). PAICS as a Potential Target for Cancer Therapy Linking Purine Biosynthesis to Cancer Progression. Life Sci..

[21] Chakravarthi B.V.S.K., Goswami M.T., Pathi S.S., Dodson M., Chandrashekar D.S., Agarwal S., Nepal S., Hodigere Balasubramanya S.A., Siddiqui J., Lonigro R.J. (2017). Expression and Role of PAICS, a De Novo Purine Biosynthetic Gene in Prostate Cancer. Prostate.

[22] Hany D., Vafeiadou V., Picard D. (2023). CRISPR-Cas9 Screen Reveals a Role of Purine Synthesis for Estrogen Receptor α Activity and Tamoxifen Resistance of Breast Cancer Cells. Sci. Adv..

[23] Meng M., Chen Y., Jia J., Li L., Yang S. (2018). Knockdown of PAICS Inhibits Malignant Proliferation of Human Breast Cancer Cell Lines. Biol. Res..

[24] Du B., Zhang Z., Di W., Xu W., Yang L., Zhang S., He G., Yang R., Wang M. (2021). PAICS Is Related to Glioma Grade and Can Promote Glioma Growth and Migration. J. Cell. Mol. Med..

[25] Li Y., Zhu L., Mao J., Zheng H., Hu Z., Yang S., Mao T., Zhou T., Cao P., Wu H. (2024). Genome-Scale CRISPR-Cas9 Screen Identifies PAICS as a Therapeutic Target for EGFR Wild-Type Non-Small Cell Lung Cancer. MedComm.

[26] Goswami M.T., Chen G., Chakravarthi B.V.S.K., Pathi S.S., Anand S.K., Carskadon S.L., Giordano T.J., Chinnaiyan A.M., Thomas D.G., Palanisamy N. (2015). Role and Regulation of Coordinately Expressed de Novo Purine Biosynthetic Enzymes PPAT and PAICS in Lung Cancer. Oncotarget.

[27] Okayama H., Kohno T., Ishii Y., Shimada Y., Shiraishi K., Iwakawa R., Furuta K., Tsuta K., Shibata T., Yamamoto S. (2012). Identification of Genes Upregulated in ALK-Positive and EGFR/KRAS/ALK-Negative Lung Adenocarcinomas. Cancer Res..

[28] Botling J., Edlund K., Lohr M., Hellwig B., Holmberg L., Lambe M., Berglund A., Ekman S., Bergqvist M., Pontén F. (2013). Biomarker Discovery in Non-Small Cell Lung Cancer: Integrating Gene Expression Profiling, Meta-Analysis, and Tissue Microarray Validation. Clin. Cancer Res..

[29] Der S.D., Sykes J., Pintilie M., Zhu C.-Q., Strumpf D., Liu N., Jurisica I., Shepherd F.A., Tsao M.-S. (2014). Validation of a Histology-Independent Prognostic Gene Signature for Early-Stage, Non-Small-Cell Lung Cancer Including Stage IA Patients. J. Thorac. Oncol..

[30] Heagerty P.J., Lumley T., Pepe M.S. (2000). Time-Dependent ROC Curves for Censored Survival Data and a Diagnostic Marker. Biometrics.

[31] The Gene Ontology Consortium (2019). The Gene Ontology Resource: 20 Years and Still GOing Strong. Nucleic Acids Res..

[32] Mariathasan S.,  Turley S.J., Nickles D., Castiglioni A., Yuen K., Wang Y., Kadel  E.E., Koeppen H., Astarita J.L., Cubas R. (2018). TGFβ Attenuates Tumour Response to PD-L1 Blockade by Contributing to Exclusion of T Cells. Nature.

[33] Hänzelmann S., Castelo R., Guinney J. (2013). GSVA: Gene Set Variation Analysis for Microarray and RNA-Seq Data. BMC Bioinf..

[34] Maeser D., Gruener R.F., Huang R.S. (2021). oncoPredict: An R Package for Predicting in Vivo or Cancer Patient Drug Response and Biomarkers from Cell Line Screening Data. Brief. Bioinform..

[35] Hu Q., Qin Y., Ji S., Shi X., Dai W., Fan G., Li S., Xu W., Liu W., Liu M. (2021). MTAP Deficiency-Induced Metabolic Reprogramming Creates a Vulnerability to Cotargeting De Novo Purine Synthesis and Glycolysis in Pancreatic Cancer. Cancer Res..

[36] Hansen L.J., Sun R., Yang R., Singh S.X., Chen L.H., Pirozzi C.J., Moure C.J., Hemphill C., Carpenter A.B., Healy P. (2019). MTAP Loss Promotes Stemness in Glioblastoma and Confers Unique Susceptibility to Purine Starvation. Cancer Res..

[37] Lv Y., Wang X., Li X., Xu G., Bai Y., Wu J., Piao Y., Shi Y., Xiang R., Wang L. (2020). Nucleotide de Novo Synthesis Increases Breast Cancer Stemness and Metastasis via cGMP-PKG-MAPK Signaling Pathway. PLOS Biol..

[38] Tabata S., Umemura S., Narita M., Udagawa H., Ishikawa T., Tsuboi M., Goto K., Ishii G., Tsuchihara K., Ochiai A. (2024). Metabolic Hallmarks for Purine Nucleotide Biosynthesis in Small Cell Lung Carcinoma. Mol. Cancer Res..

[39] Xu X., Wang L., Zang Q., Li S., Li L., Wang Z., He J., Qiang B., Han W., Zhang R. (2021). Rewiring of Purine Metabolism in Response to Acidosis Stress in Glioma Stem Cells. Cell Death Dis..

[40] Wang Q., Guan Y.F., Hancock S.E., Wahi K., Van Geldermalsen M., Zhang B.K., Pang A., Nagarajah R., Mak B., Freidman N. (2021). Inhibition of Guanosine Monophosphate Synthetase (GMPS) Blocks Glutamine Metabolism and Prostate Cancer Growth. J. Pathol..

[41] Huang Z., Xie N., Illes P., Di Virgilio F., Ulrich H., Semyanov A., Verkhratsky A., Sperlagh B., Yu S.-G., Huang C. (2021). From Purines to Purinergic Signalling: Molecular Functions and Human Diseases. Signal Transduct. Target. Ther..

[42] Keshet R., Lee J.S., Adler L., Iraqi M., Ariav Y., Lim L.Q.J., Lerner S., Rabinovich S., Oren R., Katzir R. (2020). Targeting Purine Synthesis in ASS1-Expressing Tumors Enhances the Response to Immune Checkpoint Inhibitors. Nat. Cancer.

[43] Liu X., Paila U.D., Teraoka S.N., Wright J.A., Huang X., Quinlan A.R., Gatti R.A., Concannon P. (2018). Identification of ATIC as a Novel Target for Chemoradiosensitization. Int. J. Radiat. Oncol.*Biol.*Phys..

[44] Karanika S., Karantanos T., Li L., Corn P.G., Thompson T.C. (2015). DNA Damage Response and Prostate Cancer: Defects, Regulation and Therapeutic Implications. Oncogene.

[45] Huang N., Xu C., Deng L., Li X., Bian Z., Zhang Y., Long S., Chen Y., Zhen N., Li G. (2020). PAICS Contributes to Gastric Carcinogenesis and Participates in DNA Damage Response by Interacting with Histone Deacetylase 1/2. Cell Death Dis..

[46] Fok J.H.L., Ramos-Montoya A., Vazquez-Chantada M., Wijnhoven P.W.G., Follia V., James N., Farrington P.M., Karmokar A., Willis S.E., Cairns J. (2019). AZD7648 Is a Potent and Selective DNA-PK Inhibitor that Enhances Radiation, Chemotherapy and Olaparib Activity. Nat. Commun..

[47] Wang M., Chen S., Wei Y., Wei X. (2021). DNA-PK Inhibition by M3814 Enhances Chemosensitivity in Non-Small Cell Lung Cancer. Acta Pharm. Sin. B.

[48] Sen T., Rodriguez B.L., Chen L., Corte C.M.D., Morikawa N., Fujimoto J., Cristea S., Nguyen T., Diao L., Li L. (2019). Targeting DNA Damage Response Promotes Anti-Tumor Immunity through STING-Mediated T-Cell Activation in Small Cell Lung Cancer. Cancer Discov..

[49] Lang L., Tao J., Yang C., Li W. (2022). Tumor Suppressive Role of microRNA-4731-5p in Breast Cancer through Reduction of PAICS-Induced FAK Phosphorylation. Cell Death Discov..

[50] Hosea R., Hillary S., Naqvi S., Wu S., Kasim V. (2024). The Two Sides of Chromosomal Instability: Drivers and Brakes in Cancer. Signal Transduct. Target. Ther..

[51] Agarwal S., Chakravarthi B.V.S.K., Behring M., Kim H.-G., Chandrashekar D.S., Gupta N., Bajpai P., Elkholy A., Balasubramanya S.A.H., Hardy C. (2020). PAICS, a Purine Nucleotide Metabolic Enzyme, Is Involved in Tumor Growth and the Metastasis of Colorectal Cancer. Cancers.

[52] Barfeld S.J., Fazli L., Persson M., Marjavaara L., Urbanucci A., Kaukoniemi K.M., Rennie P.S., Ceder Y., Chabes A., Visakorpi T. (2015). Myc-Dependent Purine Biosynthesis Affects Nucleolar Stress and Therapy Response in Prostate Cancer. Oncotarget.

[53] Ren B., Cam H., Takahashi Y., Volkert T., Terragni J., Young R.A., Dynlacht B.D. (2002). E2F Integrates Cell Cycle Progression with DNA Repair, Replication, and G2/M Checkpoints. Genes Dev..

[54] Bansal P., Osman D., Gan G.N., Simon G.R., Boumber Y. (2016). Recent Advances in Immunotherapy in Metastatic NSCLC. Front. Oncol..

[55] Yang Y., Yang F., Huang Z., Li Y., Shi H., Sun Q., Ma Y., Wang Y., Zhang Y., Yang S. (2023). T Cells, NK Cells, and Tumor-Associated Macrophages in Cancer Immunotherapy and the Current State of the Art of Drug Delivery Systems. Front. Immunol..

[56] Palermo B., Franzese O., Frisullo G., D’Ambrosio L., Panetta M., Campo G., D’Andrea D., Sperduti I., De Nicola F., Goeman F. (2023). CD28/PD1 Co-Expression: Dual Impact on CD8+ T Cells in Peripheral Blood and Tumor Tissue, and Its Significance in NSCLC Patients’ Survival and ICB Response. J. Exp. Clin. Cancer Res..

[57] Getu A.A., Tigabu A., Zhou M., Lu J., Fodstad Ø., Tan M. (2023). New Frontiers in Immune Checkpoint B7-H3 (CD276) Research and Drug Development. Mol. Cancer.

[58] Ren S., Tian Q., Amar N., Yu H., Rivard C.J., Caldwell C., Ng T.L., Tu M., Liu Y., Gao D. (2018). The Immune Checkpoint, HVEM May Contribute to Immune Escape in Non-Small Cell Lung Cancer Lacking PD-L1 Expression. Lung Cancer.

[59] Xu X., Chen Z., Bartman C.R., Xing X., Olszewski K., Rabinowitz J.D. (2024). One-Carbon Unit Supplementation Fuels Purine Synthesis in Tumor-Infiltrating T Cells and Augments Checkpoint Blockade. Cell Chem. Biol..

[60] Li S., Yu J., Huber A., Kryczek I., Wang Z., Jiang L., Li X., Du W., Li G., Wei S. (2022). Metabolism Drives Macrophage Heterogeneity in the Tumor Microenvironment. Cell Rep..

[61] Rowe J.H., Elia I., Shahid O., Gaudiano E.F., Sifnugel N.E., Johnson S., Reynolds A.G., Fung M.E., Joshi S., LaFleur M.W. (2023). Formate Supplementation Enhances Anti-Tumor CD8+ T Cell Fitness and Efficacy of PD-1 Blockade. Cancer Discov..

[62] Ni J., Liu Q., Xie S., Carlson C., Von T., Vogel K., Riddle S., Benes C., Eck M., Roberts T. (2012). Functional Characterization of an Isoform-Selective Inhibitor of PI3K-P110β as a Potential Anticancer Agent. Cancer Discov..

[63] He X., Chen D., Liu G., Wu Q., Zhao H., Guo D., Jiang X., Li M., Meng Y., Yin Y. (2025). PI3Kβ Functions as a Protein Kinase to Promote Cellular Protein O-GlcNAcylation and Acetyl-CoA Production for Tumor Growth. Mol. Cell.

[64] Macioszek S., Dudzik D., Bartoszewski R., Stokowy T., Lambrechts D., Boeckx B., Wozniak A., Schöffski P., Markuszewski M.J. (2023). Metabolomic and Transcriptomic Response to Imatinib Treatment of Gastrointestinal Stromal Tumour in Xenograft-Bearing Mice. Transl. Oncol..

[65] Zarou M.M., Rattigan K.M., Sarnello D., Shokry E., Dawson A., Ianniciello A., Dunn K., Copland M., Sumpton D., Vazquez A. (2024). Inhibition of Mitochondrial Folate Metabolism Drives Differentiation through mTORC1 Mediated Purine Sensing. Nat. Commun..

[66] Chou M.-C., Wang Y.-H., Chen F.-Y., Kung C.-Y., Wu K.-P., Kuo J.-C., Chan S.-J., Cheng M.-L., Lin C.-Y., Chou Y.-C. (2023). PAICS Ubiquitination Recruits UBAP2 to Trigger Phase Separation for Purinosome Assembly. Mol. Cell.

[67] Niu N., Zeng J., Ke X., Zheng W., Fu C., Lv S., Fu J., Yu Y. (2022). ATIC Facilitates Cell Growth and Migration by Upregulating Myc Expression in Lung Adenocarcinoma. Oncol. Lett..

[68] Miller J.C., Blake D.C., Herzog C.R. (2009). Adenylosuccinate Synthetase 1 Gene Is a Novel Target of Deletion in Lung Adenocarcinoma. Mol. Carcinog..

[69] Su X., Feng C., Wang S., Shi L., Gu Q., Zhang H., Lan X., Zhao Y., Qiang W., Ji M. (2021). The Noncoding RNAs SNORD50A and SNORD50B-Mediated TRIM21-GMPS Interaction Promotes the Growth of P53 Wild-Type Breast Cancers by Degrading P53. Cell Death Differ..

[70] Pey J., San José-Eneriz E., Ochoa M.C., Apaolaza I., De Atauri P., Rubio A., Cendoya X., Miranda E., Garate L., Cascante M. (2017). In-Silico Gene Essentiality Analysis of Polyamine Biosynthesis Reveals APRT as a Potential Target in Cancer. Sci. Rep..

[71] Wang L., Wang Y., Han N., Wang X., Ruan M. (2021). HPRT Promotes Proliferation and Metastasis in Head and Neck Squamous Cell Carcinoma through Direct Interaction with STAT3. Exp. Cell Res..

[72] Ali E.S., Sahu U., Villa E., O’Hara B.P., Gao P., Beaudet C., Wood A.W., Asara J.M., Ben-Sahra I. (2020). ERK2 Phosphorylates PFAS to Mediate Posttranslational Control of De Novo Purine Synthesis. Mol. Cell.

