# Supplementary material for: PAICS-Driven Purine Biosynthesis and Its Prognostic Implications in Lung Adenocarcinoma: A Novel Risk Stratification Model and Therapeutic Insights
Source: Curr Issues Mol Biol. 2025 May 16;47(5):366. doi: 10.3390/cimb47050366 (PMC12109955; doi:10.3390/cimb47050366)
Supplement: Supplementary file 1 [file cimb-47-00366-s001.zip › Supplementary materials.pdf]

Supplementary Materials

PAICS-Driven Purine Biosynthesis and Its Prognostic Implications in Lung Adenocarcinoma: A Novel Risk Stratification Model and Therapeutic Insights

1. Immunohistochemistry

Formalin-fixed paraffin-embedded (FFPE) tissue blocks from LUAD patients were used to construct tissue microarrays (TMAs). For each case, representative tumor regions were selected by a senior pathologist, and two 1.5-mm cores were extracted and arrayed into recipient paraffin blocks using a manual tissue arrayer (Beecher Instruments, Silver Spring, MD, USA). Tissue sections (5 μm thick) were deparaffinized, rehydrated, and subjected to heat-induced antigen retrieval using citrate buffer. Endogenous peroxidase activity was blocked with 3% hydrogen peroxide, followed by incubation with 5% goat serum for nonspecific blocking. Slides were incubated overnight at 4°C with a PAICS primary antibody (GTX83950, GeneTex, Irvine, CA, USA) at a 1:300 dilution. Staining intensity was semi-quantitatively scored as 0 (no staining), 1 (weak), 2 (moderate), or 3 (strong). For each core, an H-score was calculated by multiplying intensity and percentage of stained cells. Patients were stratified into high or low expression groups based on the median H-score. All samples were independently assessed by two pathologists blinded to clinical information. Discrepancies were resolved by joint review.

2. Cell Culture

Human lung cancer cell lines H460, H23, H1299, and H1975 (ATCC, Manassas, VA, USA) were maintained in RPMI-1640 medium (Gibco, Grand Island, NY, USA) supplemented with 10% fetal bovine serum (FBS) (Gibco) at 37°C in a humidified 5% CO_2 atmosphere.

3. RNA Interference and Overexpression

Cells were seeded in 6-well plates and transfected at 60–70% confluency using

Lipofectamine 3000 (Invitrogen, Carlsbad, CA, USA). Transfection complexes contained 50 nM of either a non-targeting control siRNA (NC) or PAICS-targeting siRNAs:

- siPAICS#1: 5′-GCAGUCCAAGGACCAGAUUTT-3′
- siPAICS#2: 5′-GUGUCAAGGAAGGAUAUAATT-3′

4. Quantitative Real-Time PCR (qRT-PCR)

Total RNA was isolated 48 h post-transfection using the FastRNA Pro Extraction Kit (Yishan, Shanghai, China). cDNA synthesis was performed using TransScript® One-Step gDNA Removal and cDNA Synthesis SuperMix (TransGen, Beijing, China). qRT-PCR was conducted using SYBR Green Master Mix (TransGen) on a QuantStudio 5 system (Applied Biosystems).

Primer sequences

- PAICS:
  - Forward: 5′-ACAAACAGTCTTATCGGGACCT-3′
  - Reverse: 5′-CTGCAACCCACTCAAAGTTTTTC-3′
- ACTB (β-actin, internal control):
  - Forward: 5′-AGGATTCCTATGTGGGCGAC-3′
  - Reverse: 5′-ATAGCACAGCCTGGATAGCAA-3′

Gene expression was normalized to β-actin (ACTB) using the $2^{-\Delta\Delta Ct}$ method.

5. Western Blotting

Cells were lysed 72 h post-transfection in RIPA buffer (Pulilai Gene Technology, Beijing, China) containing protease inhibitors. Protein concentrations were determined using a bicinchoninic acid (BCA) assay (Thermo Fisher Scientific). Lysates (30 μg/lane) were separated by SDS-PAGE, transferred onto PVDF membranes, and probed with:

- Anti-PAICS antibody (1:1000, GeneTex)
- Anti-α-Tubulin antibody (1:2000, #2144, Cell Signaling Technology)

HRP-conjugated secondary antibodies (#7076S, Cell Signaling Technology) and ECL substrate (Bio-Rad) were used for detection.

6. CCK-8 Cell Proliferation Assay

Cells (3,000/well) were plated in 96-well plates and assessed daily for 5 days using CCK-8 reagent (Dojindo, Kumamoto, Japan). Absorbance at 450 nm was measured using a microplate reader (BioTek).

7. Colony Formation Assay

Transfected cells (1,000/well) were seeded into 6-well plates and cultured for 14 days. Colonies (>50 cells) were stained with 0.1% crystal violet and counted using ImageJ software.

8. Transwell Migration Assay

Cells (4×10^4/well) suspended in serum-free medium were seeded into Transwell chambers (8-μm pore size, Corning). After 28 h, migrated cells were fixed, stained, and quantified.

9. Wound Healing Assay

Confluent cell monolayers in 96-well plates were scratched using a WoundMaker™ (Essen BioScience). Closure was monitored over 48 h in serum-free medium using the IncuCyte S3 system (Sartorius).

10. Drug Sensitivity Assay

Cells were seeded into 96-well plates at a density of 3,000–5,000 cells per well and allowed to adhere overnight. The following day, cells were treated with serial dilutions of the indicated compounds, including, AZD6482 and Imatinib, across a range of concentrations (0.01–100μM). Each concentration was tested in triplicate. After 72 hours of treatment, cell viability was assessed using the Cell Counting Kit-8 (CCK-8, Dojindo) according to the manufacturer's instructions. Absorbance was measured at 450 nm.

11. **Statistical Analysis**

Data are presented as mean ± SD from ≥3 independent experiments. Statistical analyses were conducted using R v4.0.5 and GraphPad Prism v8.0.2.

- Survival curves were generated using Kaplan-Meier analysis with log-rank tests.
- Categorical variables were compared using χ^2 tests.
- Continuous variables were analyzed using Mann-Whitney U tests for non-parametric data and unpaired two-tailed Student's t-tests for normally distributed data.
- Model construction: LASSO regression was applied to select purine biosynthesis-related genes (PBRGs) while minimizing overfitting. The regularization parameter (λ) was optimized using 10-fold cross-validation.
- Cox regression: Multivariate Cox regression was performed to evaluate PBRS as an independent prognostic factor, reporting hazard ratios (HR) with 95% confidence intervals (CI).

Supplementary Figure Legends

Figure S1. Prognostic Significance of Signature Genes in TCGA-LUAD

Kaplan-Meier survival curves for (A) PAICS, (B) ATIC, (C) GMPS, (D) ADSS1, (E) APRT, and (F) HPRT in TCGA-LUAD.

Figure S2. Clinicopathological Characteristics

Clinicopathological characteristics of high-risk vs. low-risk LUAD patients
