# Supplementary figures and images for: PAICS-Driven Purine Biosynthesis and Its Prognostic Implications in Lung Adenocarcinoma: A Novel Risk Stratification Model and Therapeutic Insights
Source: Curr Issues Mol Biol. 2025 May 16;47(5):366. doi: 10.3390/cimb47050366 (PMC12109955; doi:10.3390/cimb47050366)

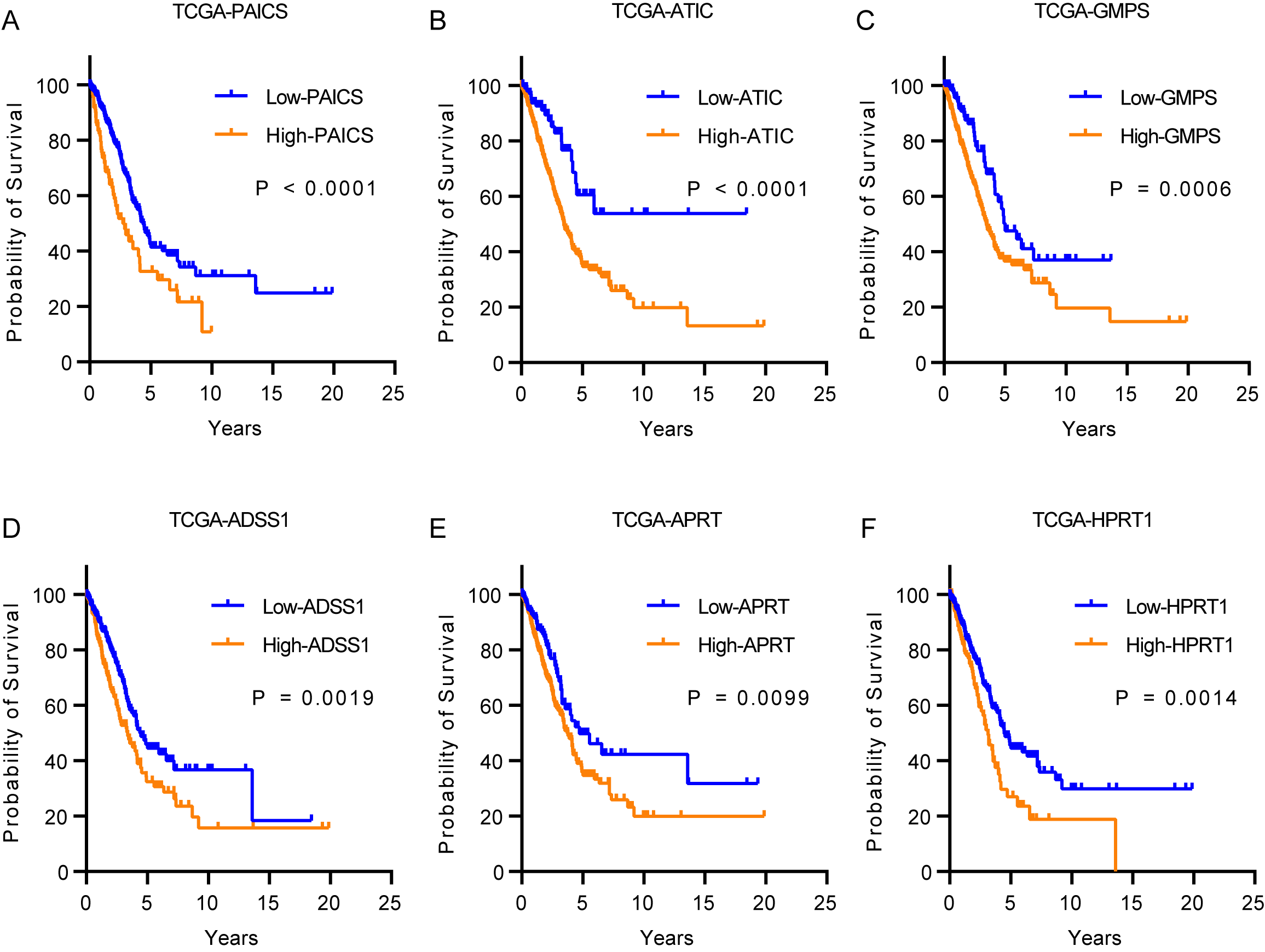

Supplement: Supplementary file 1 [file cimb-47-00366-s001.zip › Supplementary Figure S1.tif]

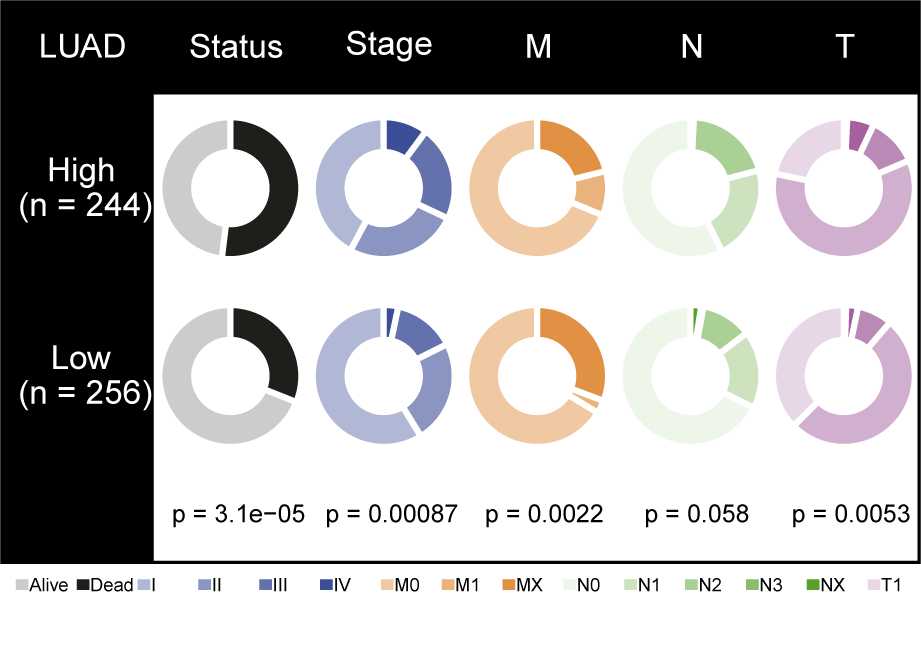

Supplement: Supplementary file 1 [file cimb-47-00366-s001.zip › Supplementary Figure S2.tif]
